# Plant–fungi interactions in *Marchantia polymorpha* are associated with horizontal gene transfer and terpene metabolism

**DOI:** 10.1073/pnas.2532723123

**Published:** 2026-02-04

**Authors:** Karima El Mahboubi, Chloé Beaulieu, Baptiste Castel, Cyril Libourel, Nathanaël Jariais, Emilie Amblard, Fabian van Beveren, Jean Keller, Yves Martinez, Jessica M. Nelson, Maxime Bonhomme, Christophe Jacquet, Pierre-Marc Delaux

**Affiliations:** ^a^Laboratoire de Recherche en Sciences Végétales, Université de Toulouse, CNRS, Institut National Polytechnique-AgroToulouse, Castanet-Tolosan 31320, France; ^b^TRI-FRAIB Imaging Platform Facilities, FRAIB, Université de Toulouse, CNRS, Castanet-Tolosan 31320, France; ^c^Natural History Museum, University of Oslo, Oslo 0562, Norway

**Keywords:** Evo-MPMI, Marchantia, plant evolution

## Abstract

The liverwort *Marchantia polymorpha* has emerged as a model for studying plant immunity in bryophytes, providing unique insights into conserved defense mechanisms across land plants. By contrast, Marchantia-specific immune mechanisms remained largely underexplored. In this study, we investigated the genetic basis of quantitative resistance in *M. polymorpha* against the fungal pathogen *Colletotrichum nymphaeae*, a naturally occurring compatible parasite. Through a combination of phenotypic, cytological, and transcriptomic approaches, combined with genome-wide association studies (GWAS), we identified key defense-related genes and pathways. Leveraging the biological and genetic variability present in a collection of natural *M. polymorpha* accessions, we highlight the role of horizontally transferred microbial-like terpene synthase genes, which may contribute to the exceptional terpene diversity of liverworts and potentially play a role in pathogen resistance. GWAS uncovered candidate loci associated with resistance traits, implicating both core immune components and specialized metabolic pathways. Transcriptomic analyses performed on two accessions with contrasting phenotypes after inoculation with *C. nymphaeae* revealed the upregulation of accession-specific and horizontally acquired genes. These results provide insights into the specific molecular underpinnings of bryophyte immunity and underscore the evolutionary significance of horizontal gene transfer and specialized metabolites in shaping plant–pathogen interactions.

## Significance

The study of plant immunity has predominantly focused on flowering plants, nonvascular plants being largely unexplored. Here, we find immune mechanisms operating in the nonvascular plant model Marchantia by comparing the genomes and immune competence of multiple wild populations. Among the genes associated with resistance, we identified enzymes which have been transferred from microorganisms into the genome of the most recent common ancestor of the nonvascular plants. Although further functional tests are required to establish the contribution of these enzymes to defense, this finding sheds light on the evolutionary importance of horizontal gene transfer in shaping the diversity of plant immunity. This work emphasizes the value of exploring underrepresented plant lineages to uncover biological processes.

Half a billion years ago, plants transitioned from an aquatic to a terrestrial environment (1). This habitat shift required the evolution of adaptations to abiotic challenges such as nutrient scarcity or UV stress and drought (2). Simultaneously, mirroring the arms race observed in extant ecosystems, biotic pressures from emerging pathogens likely drove the evolution and diversification of plant immune responses (3).

Extant land plant species are divided into two main lineages, the tracheophytes (vascular plants, which include flowering plants), and the bryophytes that include liverworts, mosses, and hornworts (4). These two lineages diverged from each other early after the colonization of land (4), allowing to reconstruct the biology of the first land plants by comparing extant species belonging to the tracheophytes and bryophytes to identify similarities and homologies (5). This allowed, for instance, the identification of an ancestral biosynthesis pathway for the plant cuticle (6), the demonstration of the ancestral nature of symbiotic interactions (7) and the occurrence of a shared program for the development of epidermal structures across land plants (8).

While physiological and molecular mechanisms involved in plant immunity have been extensively studied in flowering plants (9), our understanding of defense mechanisms in bryophytes remains limited. Phylogenetic surveys across the diversity of land plants (10) and targeted functional analyses in model bryophytes have revealed the conservation of several immune components, including the involvement of Lysin-motif Receptor-like Kinase (LysM-RLK) receptors for the perception of fungal pathogens and the activation of downstream MAP-Kinases (11, 12), the activation of cell-death by the N-terminal domain of NLRs (13), NPR1 and salicylic acid (SA) as key elements in hormonal immune signaling (14), or phenylpropanoids as major components of plant defenses against pathogenic oomycetes (15). In contrast to these immune features conserved in tracheophytes and bryophytes, angiosperm specificities, such as the plasma membrane-located pattern recognition receptors FLS2 or EFR (16), or the biosynthesis of jasmonic acid are absent from bryophyte immunity (17). Bryophytes also display unique features, including atypical immune receptor configurations such as NLRs harboring α/β hydrolase or protein kinase domains in their N-terminal domain (10, 13), highlighting the independent evolution of defense mechanisms over the past 400 My in the two main land plant lineages. So far, the discovery and identification of specific immune mechanisms have remained underexplored in bryophytes, with the notable exception of the defensive role played by oil bodies against herbivores (18). Uncovering the origins and genetic basis of bryophyte-specific immune mechanisms is essential to understanding how plant immune systems evolved across the green lineage. The liverwort *Marchantia polymorpha* offers unique advantages for such studies. This plant is a well-established model bryophyte, genetically tractable (19), with a sequenced genome (16) and a diversity of compatible interactions with well-described angiosperm pathogens including oomycetes (20), bacteria (21), fungi (22, 23), and viruses (24). Transcriptomic analyses of the plant’s response to pathogenic oomycete (15) and bacteria (25) were also performed, highlighting evolutionarily conserved mechanisms among land plants. A collection of wild-collected accessions from Europe, the United States, and Japan is also available for the three *M. polymorpha* subspecies (26). The genomes of these accessions have been resequenced and compared, leading to the identification of Single Nucleotide Polymorphisms (SNPs) at the species level, making *M. polymorpha* amenable for genome-wide association studies—GWAS (26).

Here, we leveraged these resources to investigate the genetic basis of quantitative resistance in *M. polymorpha* against the *M.* polymorpha-isolated fungal pathogen *Colletotrichum nymphaeae*. We first conducted complementary phenotypic, cytological, and transcriptomic analyses to characterize a natural pathosystem between two *M. polymorpha* accessions with contrasting phenotypes following *C. nymphaeae* inoculation. Additionally, we utilized the genetic diversity within the *M. polymorpha* collection to perform the first genome-wide association study with a bryophyte. Our findings reveal both conserved and lineage-specific immune mechanisms, highlighting a potential role for terpene metabolism and horizontally transferred genes in the adaptation to this pathogen.

## Results

### *Colletotrichum nymphaeae* Is a Compatible Parasite of *Marchantia Polymorpha* Tak-1.

**Fig. 1.**
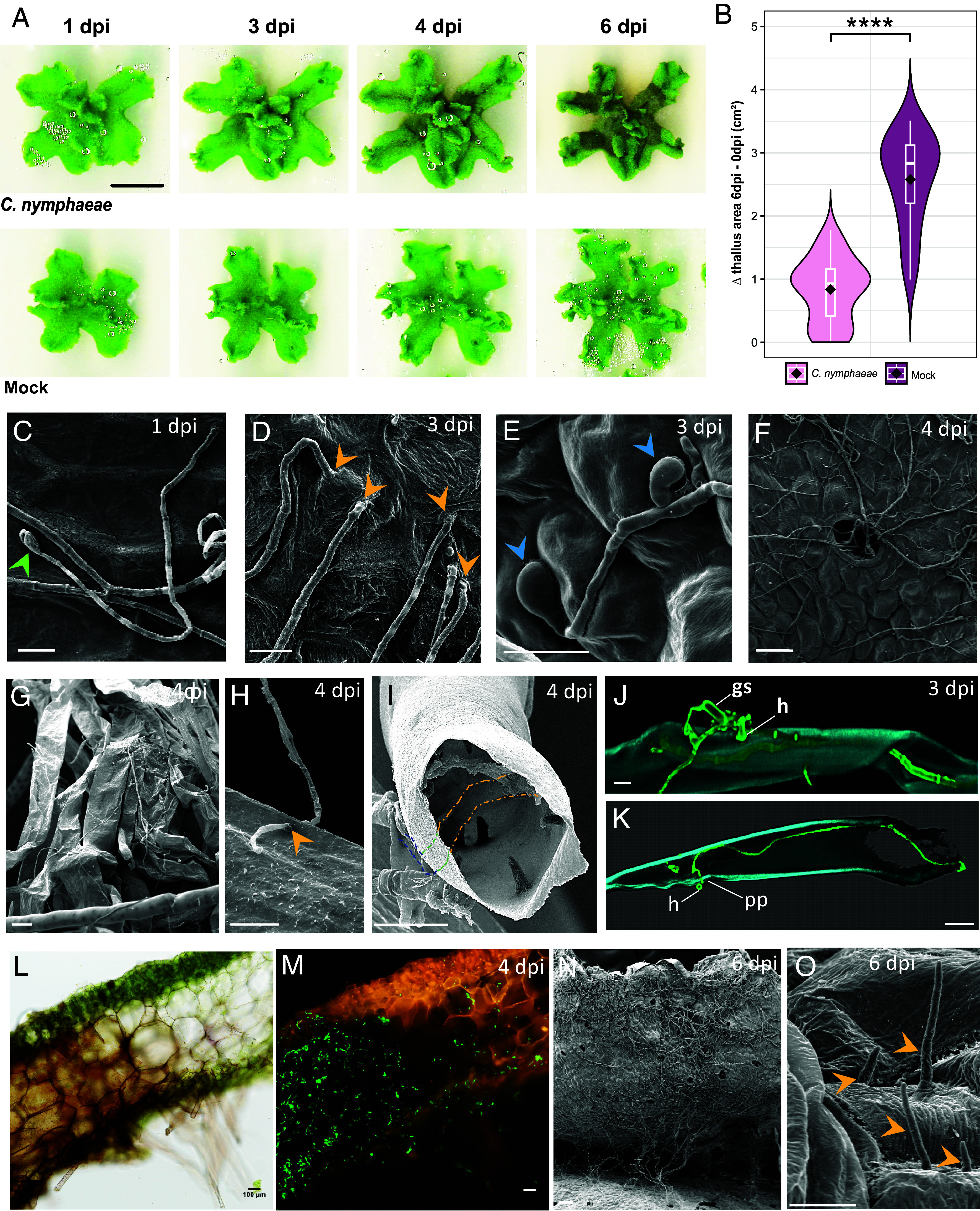
*C. nymphaeae* completes its biological cycle in *M. polymorpha*. (*A*) Phenotypes of Tak-1 thalli from 0 to 6 dpi, after *C. nymphaeae* or mock inoculation (bar = 1 cm). Contrast and brightness were increased by 40% from the raw image. (*B*) Boxplots and violin plots from n = 68 measured samples. Each dot represents the difference between the surface of the thallus at 6 dpi and the surface measured of the thallus just before inoculation, for one sample, in inoculated (*Left*) or mock (*Right*) conditions. (****) indicates a *P*-value of 6.436e-13 from a Welch’s *t* test. (*C*) Germinated spore (green arrowhead) at 1 dpi; (*D*) Following germination, direct penetration of fungal hyphae through the thallus cuticle (yellow arrowheads). (*E*) Formation of appressorium-like structures (blue arrowheads); (*F*) Entry of hyphae through air pores. (*C*–*E*; bar = 10 µm; bar = 50 µm in *F*). From 4 dpi, hyphae can also be observed, crawling on the surface of rhizoids, (*G* and *H*) or inside them, both in pegged (*I*) or in smooth (*J* and *K*) rhizoids, following a direct (*H*) penetration (yellow arrowhead), or using hyphopodia (*J* and *K*). Panel *I* shows a SEM image with dashed lines marking the hyphae outside (blue) or inside (green and yellow) the pegged rhizoid, highlighting both extracellular and intracellular growth. In (*J*) hyphae labeled with WGA-FITC (green) develop on the rhizoid surface, forming hyphopodia and initiating penetration; (*K*) corresponds to a confocal cross-section showing an intracellular hypha entering from the rhizoid surface stained in blue by calcofluor. gs: germinated spore, h: hyphopodium, pp: penetration peg. (Bars = 10 µm in *G*, *H*, and *I* and 50 µm in *J* and *K*). At 4 dpi macerated and brown tissues are observed in brightfield (*L*), with abundant WGA-FITC-labeled hyphae visible under fluorescence (*M*). At 6 dpi, thalli are heavily colonized (*N*), and at this late time point, *setae* (yellow arrowheads) emerge from the thalli (*O*). *L* and *M* are the same 100 µm-thick sections of infected thallus observed 6 dpi in brightfield (*L*) or under excitation with blue light (*M*). (Bars = 50 µm in *L* and *M*; 100 µm in *N* and 10 µm in *O*).

To explore the immune mechanisms of *M. polymorpha*, we selected one of the most pathogenic fungi identified in a previous survey of endophytes isolated from natural populations of *M. polymorpha* (23). Previously referred to as *Colletotrichum sp.*, we identified this strain as *C. nymphaeae* based on six classical markers commonly used for *Colletotrichum sp*. taxonomy (27, see *Online Methods* section). *C. nymphaeae*, widely known as a hemibiotrophic pathogen of strawberries, belongs to the *Colletotrichum acutatum* complex and is the causal agent of anthracnose fruit rot, characterized by dark-colored, sunken lesions (28). To characterize the interaction between the model accession *M. polymorpha* Tak-1 and *C. nymphaeae*, a 10 µL droplet of conidia (10^4^ spores/mL) was deposited in the center of each thallus and a kinetics of infection was monitored over 6 d. The impact of *C. nymphaeae* on *M. polymorpha* Tak-1 development was monitored by scanning the plants every 24 h and comparing inoculated and mock-inoculated plants (Fig. 1). No symptoms were observed during the first two days following inoculation (Fig. 1*A*). The first necrotic spots occurred at 3 d post inoculation (dpi) at the inoculation point (Fig. 1*A*), with brown tissues spreading throughout the thalli, resulting in fully macerated plants at 6 dpi (Fig. 1*A*). In some thalli, meristematic tissues remained green, an observation reminiscent of results described with angiosperm pathogens inoculated on *M. polymorpha* (20, 21, 29). Differences between the thallus sizes measured before inoculation and at 6 dpi for each mock- or *C. nymphaeae*-inoculated thallus revealed a significant negative impact of *C. nymphaeae* on the growth of *M. polymorpha Tak-1* thalli (Welch’s two-sample *t* test, *P*-value = 6.436 × 10^−13^) (Fig. 1*B*).

Scanning Electron Microscopy (SEM) was conducted on samples collected 2, 3, 4, and 6 dpi, to better describe the infection. Germination of the *C. nymphaeae* conidia typically occurred within the first day after inoculation (Fig. 1*C*). Fungal colonization was observed through diverse strategies, including direct penetration (Fig. 1*D*), development of dome-shaped appressorium-like structures (Fig. 1*E*), and colonization via air pores (Fig. 1*F*). At 4 dpi, the mycelium was clearly visible on the thallus surface (Fig. 1 *G*, *H*, and *J*), and inside the rhizoids (Fig. 1 *I* and *K*). Rhizoid colonization occurred either directly (Fig. 1*H*) or via the formation of hyphopodia (Fig. 1 *J* and *K*). Based on symptoms, the stage transition from biotrophic to necrotrophic takes place between 2 and 3 dpi. At 4 dpi fungal hyphae massively colonized brown, dead or dying cells (Fig. 1 *A*, *L*, and *M*). Finally, by 6 dpi, the macerated thalli were heavily covered with mycelium (Fig. 1 *A* and *N*). SEM analysis also revealed emerging setae, fungal structures that precede the formation of the asexual fruiting body structures (acervuli) (Fig. 1*O*).

Altogether, these results indicate that *C. nymphaeae* is a natural hemibiotrophic fungal parasite of *M. polymorpha*, capable of completing its biological cycle on the *M. polymorpha* Tak-1 accession during a fully compatible interaction. While the *Colletotrichum genus* includes species with diverse lifestyles ranging from necrotrophy to endophytism, the infection dynamics observed here are consistent with the hemibiotrophic behavior reported for *C. nymphaeae* on angiosperms (30).

### *M. polymorpha* Displays Quantitative Resistance to *Colletotrichum nymphaeae*.

**Fig. 2.**
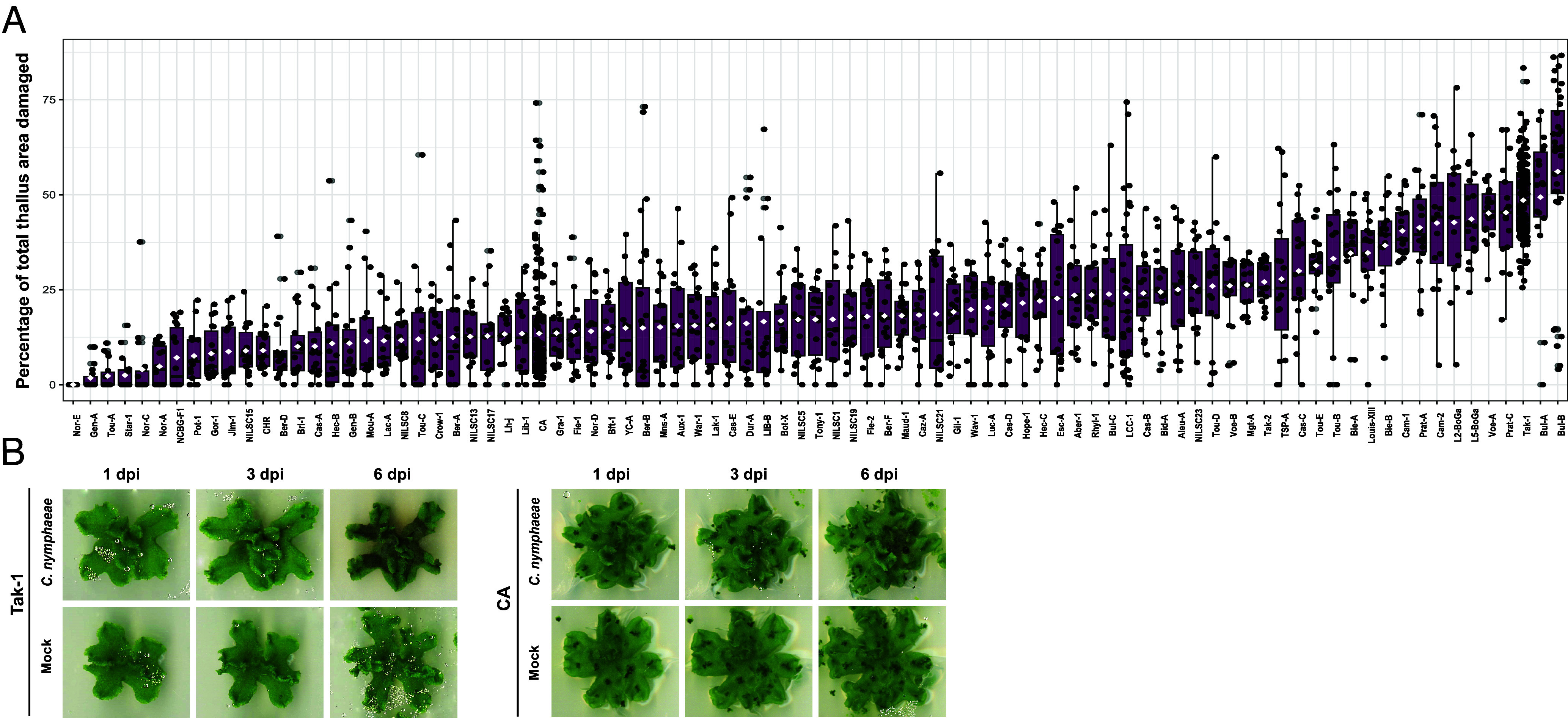
*M. polymorpha* exhibits quantitative resistance to *Colletotrichum nymphaeae*. (*A*) Percentage of total thallus area damaged, measured at 6 dpi for 87 accessions. For each accession, excluding the two internal controls Tak-1 and CA, 15 to 18 three-week-old thalli were inoculated. (*B*) Disease symptoms on *M. polymorpha* Tak-1 (*Left*) and CA (*Right*) thalli inoculated with *C. nymphaeae*, at 1, 3, and 6 dpi.

Given that *C. nymphaeae* is a naturally occurring compatible pathogen of *M. polymorpha*, we hypothesized that diverse levels of resistance might exist across *M. polymorpha* populations. To test this hypothesis, we took advantage of the recently characterized collection of *M. polymorpha* accessions (26) and selected a set of 87 easily propagated accessions to be assayed against *C. nymphaeae*. For each accession, 36 freshly collected gemmae were grown axenically on a sugar-free medium for 3 wk before being inoculated with *C. nymphaeae* or left untreated. Symptoms were monitored over time as described in the Methods section. Since the first symptoms are visible as early as 3 dpi in most accessions and showed little to no progression after 6 dpi, the latter time point was retained for symptoms quantification. The distribution of the symptom proportion (i.e., the ratio of symptomatic area–brown_area - to the total thallus area–thallus_area -) for the 87 accessions revealed a high variability of responses to *C. nymphaeae* (Fig. 2*A*). Indeed, the average proportion of symptoms per accession at 6 dpi ranged from 0 to 59% for the most resistant and susceptible accessions, respectively. In the most resistant accessions, such as Nor-E, most thalli exhibited no symptoms at 6 dpi, suggesting complete resistance to *C. nymphaeae*. For other resistant accessions, such as CA, symptoms often remained restricted to the inoculation point in the middle of the thallus (Fig. 2*B*). By contrast, less resistant accessions displayed significant symptom development (*SI Appendix*, Fig. S1). The reference accession *M. polymorpha* Tak-1 belongs to the group of the most susceptible accessions (Fig. 2*B*).

We conclude from this phenotypic screen that *M. polymorpha* exhibits quantitative resistance to *C. nymphaeae.*

### *Marchantia polymorpha* Mounts a Generic Transcriptional Response to Filamentous Pathogens.

To investigate how *M. polymorpha* responds to infection by *C. nymphaeae*, we performed an RNA-seq analysis on the susceptible accession *M. polymorpha* Tak-1 comparing the transcriptional profiles of 3-wk-old thalli inoculated with water (mock) or with a suspension of *C. nymphaeae* for three and six days (31). Differential expression analysis (adjusted *P*-value ≤ 0.05 and |log_2_ fold change| ≥ 1) was conducted for mock-treated versus inoculated thalli at 3 dpi, as RNA extraction from 6 dpi samples was compromised due to excessive maceration.

**Fig. 3.**
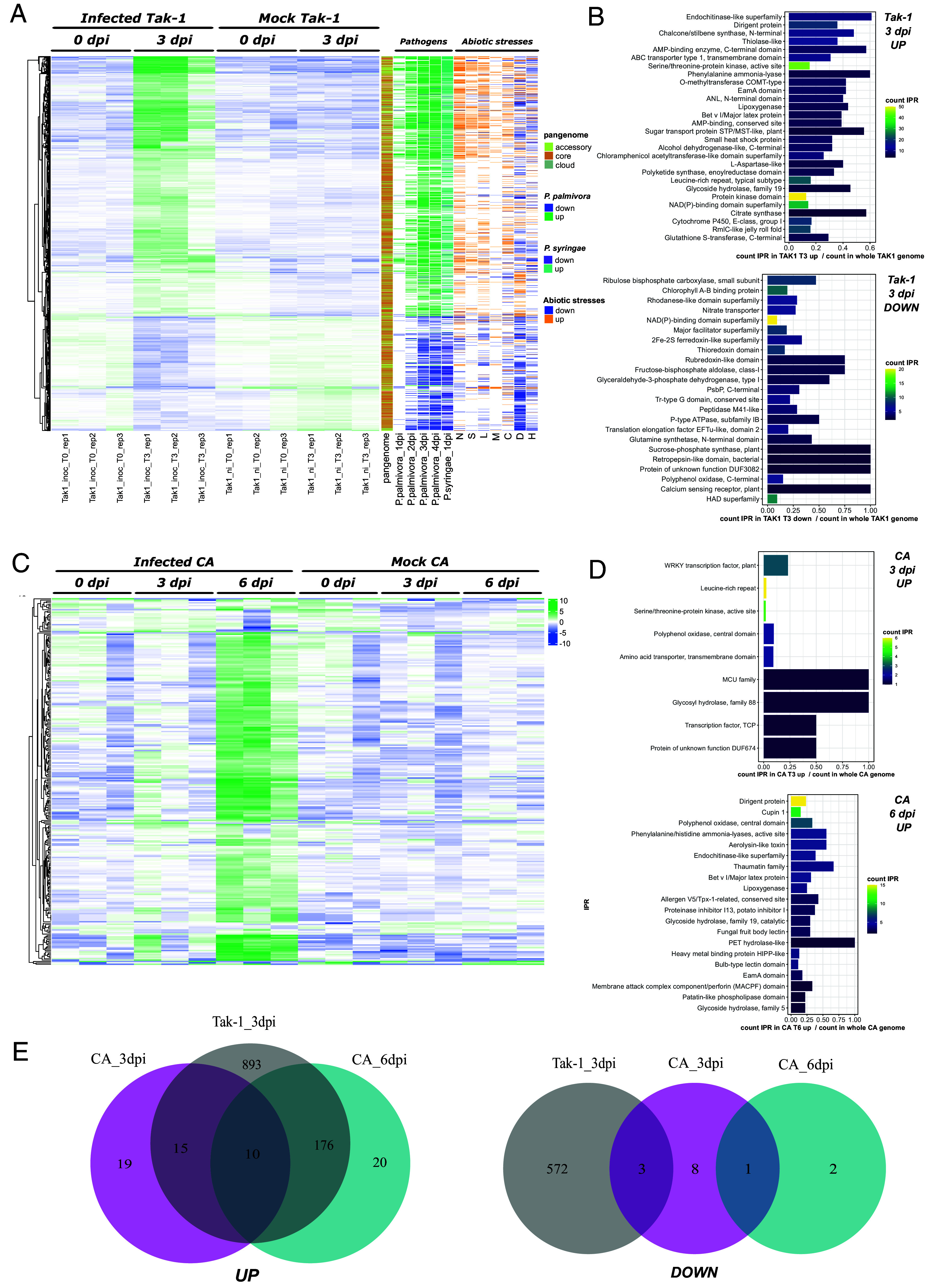
The *Marchantia polymorpha* ssp. *ruderalis* Tak-1 and CA accessions display striking temporal and functional discrepancies in their differential gene expression in response to *C. nymphaeae*. (*A*) Hierarchical clustering of Tak-1’s significantly differentially expressed genes during *C. nymphaeae* infection (adjusted *P* ≤ 0.05; |log_2_ fold change| ≥ 1) at 0 and 3 d post inoculation (dpi). Variance-stabilized row-centered counts are shown. Additional information on these genes is shown on the right side of the heatmap: the pangenomic compartment of the genes, differential expression during infection with *P. palmivora* (adjusted *P*-value ≤ 0.05; |log_2_ fold change| ≥ 1), and differential expression under different abiotic stresses (N = nitrogen deficiency, S = salt, L = light, M = mannitol, C = cold, D = dark, H = heat). (*B*) Functional enrichment of IPR terms in Tak-1’s up- and downregulated genes at 3 dpi (enrichment cutoff 0.01). Redundant IPR terms were discarded to improve readability. (*C*) Hierarchical clustering of CA’s significantly differentially expressed genes during *C. nymphaeae* infection (adjusted *P* ≤ 0.05; |log_2_ fold change| ≥ 1) at 0, 3, and 6 d post inoculation (dpi). Variance-stabilized row-centered counts are shown. (*D*) Functional enrichment of IPR terms in CA’s upregulated genes at 3 and 6 dpi (enrichment cutoff 0.01). Redundant IPR terms were discarded to improve readability. (*E*) Comparison of differentially regulated genes in Tak-1 and CA at different time points post inoculation (upregulated genes on the left side, downregulated genes on the right side). These Venn diagrams only represent the genes for which a gene-to-gene correspondence between CA and Tak-1 could be determined.

This analysis revealed strong transcriptional reprogramming in *M. polymorpha* Tak-1 following fungal infection (Fig. 3*A* and Dataset S1) with 1,266 genes upregulated and 575 genes downregulated at 3 dpi. Functional enrichment analysis of *M. polymorpha* Tak-1 upregulated genes identified InterPro domains associated with mechanical defenses, such as dirigent proteins and the O-methyltransferase COMT-type domains, which may be involved in cell-wall component biosynthesis. Domains associated with specialized metabolism, including phenylpropanoid and flavonoid biosynthesis, such as the phenylalanine ammonia-lyase and the chalcone/stilbene synthase domains, were also enriched (Fig. 3*B*). Additionally, domains implicated in general plant defenses and related signaling, such as chitinases, Bet v I (PR-proteins), and lipoxygenases (oxylipin pathway), were significantly enriched (Fig. 3*B* and Dataset S2).

Interestingly, genes most intensely upregulated in response to *C. nymphaeae* were predominantly accessory genes from the *M. polymorpha* ssp. *ruderalis* pangenome (63% of accessory genes in DEGs with a logFC ≥ 5 versus 33% of accessory genes in DEG with a log FC ≤5, chi-square test *P*-value of 2.55 × 10^−12^). These results support the hypothesis that the accessory compartment of the pangenome plays an adaptive role in *M. polymorpha* immunity (26).

To determine whether this transcriptional reprogramming is specific to *C. nymphaeae* or part of a broader response, we compared our data with differential expression analyses of *M. polymorpha* infected by the oomycete *Phytophthora palmivora* (15) and by the bacterial pathogen *Pseudomonas syringae* B728A (25). Among the genes up-downregulated in response to *C. nymphaeae* inoculation, 80%/64% and 57%/48%% were also differentially regulated in Tak-1 following infections by *P. palmivora*/*P. syringae,* respectively, suggesting a common transcriptional program in Marchantia, mainly in response to filamentous pathogens (Dataset S3).

Among the commonly up-regulated functions in response to filamentous pathogens, both in *M. polymorpha* and *N. benthamiana*, are isoprenoid biosynthesis and peroxisome-related processes (Dataset S4). Notably, some Tak-1 genes up-regulated in response to *C. nymphaeae* (this study), to *P. palmivora* (15), and to *P. syringae* (25) were also associated with climatic variation in a genome–environment association study (26). These include genes such as *MpNBS-LRR11* (Mp4g08790), *LURP1* (Mp4g08800), or *MpLOX5* (Mp1g21930), which were also differentially regulated in response to abiotic stresses (Dataset S3). This observation suggests their involvement in multiple stress responses or reflects a close relationship between adaptation to climatic conditions and pathogen pressure.

We conclude that the *M. polymorpha* Tak-1 transcriptional response to *C. nymphaeae* includes a generic immune response to microbial pathogens, as well as pathogen-specific transcriptional adjustments, partially overlapping with general stress responses in *M. polymorpha*.

### Quantitative Resistance Is Associated with a Specific Transcriptomic Response.

To assess the transcriptional differences between accessions with varying levels of resistance, a differential expression analysis was performed on one of the most resistant accessions, *M. polymorpha* CA, isolated in the United States (26), on samples collected at three and six dpi. At three dpi, only 44 genes were significantly upregulated, and 12 downregulated (Fig. 3*C*). At six dpi, 221 genes were significantly upregulated and three genes downregulated (adjusted *P*-value ≤ 0.05 and |log_2_ fold change| ≥ 1) (Fig. 3*C* and Dataset S5).

Compared to *M. polymorpha* Tak-1, the *M. polymorpha* CA accession exhibits a delayed transcriptional response to the pathogen, with only a few genes differentially expressed at 3 dpi (Fig. 3*C*), possibly indicating the presence of constitutive defense mechanisms in CA that delay the penetration of the pathogen and/or its perception by the plant. The limited transcriptional upregulation at 3 dpi was enriched in domains from transcription factors (WRKY and TCP) and other signaling components (Leucine-rich repeats, kinases) commonly involved in plant—pathogen interactions (Fig. 3*D* and Dataset S6). Interestingly, one of the enriched domains, a glycoside hydrolase 88 (GH88), is typically described in bacteria and fungi. Searching for this domain across plant lineages revealed its presence in mosses, liverworts, lycophytes, and ferns, but not in hornworts nor in seed plants. Further phylogenetic analysis suggests a potential horizontal gene transfer (HGT) from fungi to the common ancestor of land plants, followed by subsequent losses in some lineages (*SI Appendix*, Fig. S2).

At 6 dpi, the transcriptional response of *M. polymorpha* CA became more pronounced, resembling the *M. polymorpha* Tak-1 response at 3 dpi. Enriched domains include dirigent proteins, PR10 (Bet v I/Major latex protein domain), lipoxygenases, phenylalanine ammonia lyases, polyphenol oxidases, and chitinases. Other well-known defense-related protein families, such as various pathogenesis-related (PR) proteins (PR1: Allergen V5/Tpx-1-related, PR5: Thaumatin family, PR6: proteinase inhibitor, PR15: cupins) were also enriched. The Membrane Attack Complex Component/perforin (MACPF) domain, conserved across prokaryotes and eukaryotes was also present. MACPF proteins are involved in immune responses by forming pores in the membranes of pathogens (32). Only two MACPF genes have been described in *Arabidopsis* and are involved in immune response regulation in *Arabidopsis* (33, 34). Additionally, fungal fruit body lectins, originating from an HGT event in the ancestor of land plants (26), were enriched in the *M. polymorpha* CA response to *C. nymphaeae*. To enable a more precise comparison between the differentially expressed genes in the two accessions, a gene-to-gene correspondence was established between the two genomes (*SI Appendix*). The overlap analysis (Fig. 3*E*) revealed ten genes commonly upregulated at all time points in both accessions. These genes encode for proteins including transcription factors (MpWRKY3 and 7), secondary metabolite synthases (MpPAL7, MpPKS/CHS11, and MpPPO3), a peroxidase (PR9b), and an oxylipin synthesis enzyme (LOX1). Among the 18 genes uniquely upregulated in CA, several encode proteins associated with Aerolysin/ETX pore-forming domains (four genes), cupins (three genes), and cytochrome P450 enzymes (Dataset S3).

These results indicate a delayed yet targeted transcriptional response in *M. polymorpha* CA, consistent with its resistance phenotype, characterized by limited early reprogramming involving a core set of defense genes along with the recruitment of accession-specific immune components.

### Identification of Genetic Loci Associated with Resistance/Susceptibility Variation by GWAS.

Given the diversity of phenotypic responses to inoculation with *C. nymphaeae* across the panel of *M. polymorpha* accessions, and the distinct transcriptional responses between the Tak-1 and CA accessions, we conducted a GWAS to explore the genetic basis of these responses. The association analysis was performed on 77 phenotyped accessions of *M. polymorpha ssp. ruderalis*, focusing on three traits: the thallus area of inoculated plants at 6 dpi, the effect of the fungus on plant growth, and the area of symptoms (browning) at 6 dpi (Dataset S7). Significant genomic loci were identified for each trait (Dataset S8).

**Fig. 4.**
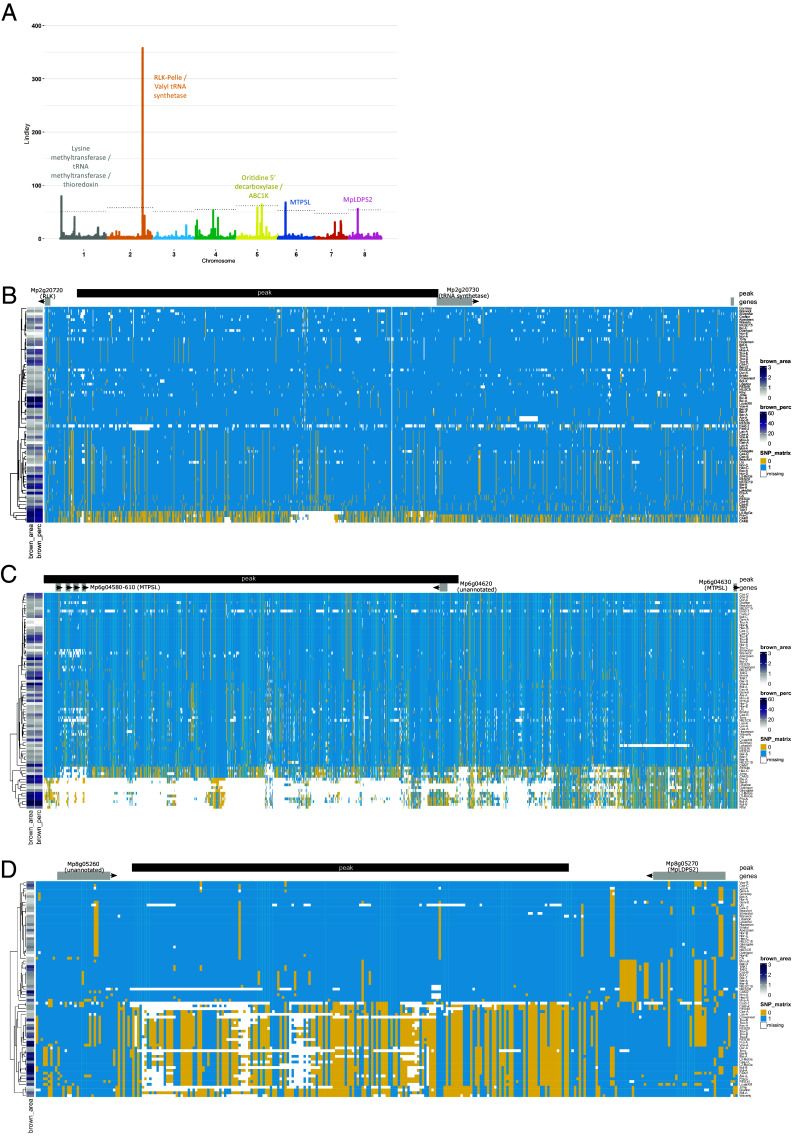
Identification of loci associated with phenotypic differences among *M. polymorpha* ssp. *ruderalis* accessions in response to *C. nymphaeae*. (*A*) Manhattan plot of the GWAS results on the area of symptoms (brown area) on the thallus of inoculated plants at 6 dpi. This plot and the previous one result from a classical GWAS analysis performed with GEMMA, followed by the use of the local score technique on the SNP *P*-values, to amplify the signal between SNPs in LD. The result of this process is a Lindley value that has been used instead of *P*-value to plot the y-values of the Manhattan plots. The dotted lines represent the significance thresholds for each chromosome (resampling thresholds from the local score method). (*B*) Haplotype block illustration of the genomic region on chromosome 2 associated with both the brown area of the symptoms (brown_area) and the ratio of symptoms to the total area of the thallus (brown_perc) in *M. polymorpha* ssp. *ruderalis*. The association peak (black rectangle) is flanked by two protein-coding genes: an RLK-Pelle (Mp2g20720) and a tRNA synthetase (Mp2g20730). (*C*) Haplotype block illustration of the genomic region on chromosome 6 associated with both the brown area of the symptoms (brown_area) and the ratio of symptoms to the total area of the thallus (brown_perc) in *M. polymorpha* ssp. *ruderalis*. The association peak (black rectangle) is overlapping four MTPSL genes (Mp6g04580-Mp6g04610) and an unannotated gene (Mp6g04620). A fifth MTPSL gene (Mp6g04630) is located downstream of the peak. (*D*) Haplotype block illustration of the genomic region on chromosome 8 associated with the brown area of the symptoms (brown_area) in *M. polymorpha* ssp. *ruderalis*. The association peak (black rectangle) is flanked by two protein-coding genes: an unannotated gene (Mp8g05260) and the MpLDPS2 gene (Mp8g05270). For the three haplotype block illustrations, the gradient on the left side of the figure represents the values of the phenotypes of each accession that appear to be associated with the represented region. The main matrix represents the allelic status of the SNPs in this region for each accession: major allele (blue) or minor allele (yellow), missing information (white).

**Fig. 5.**
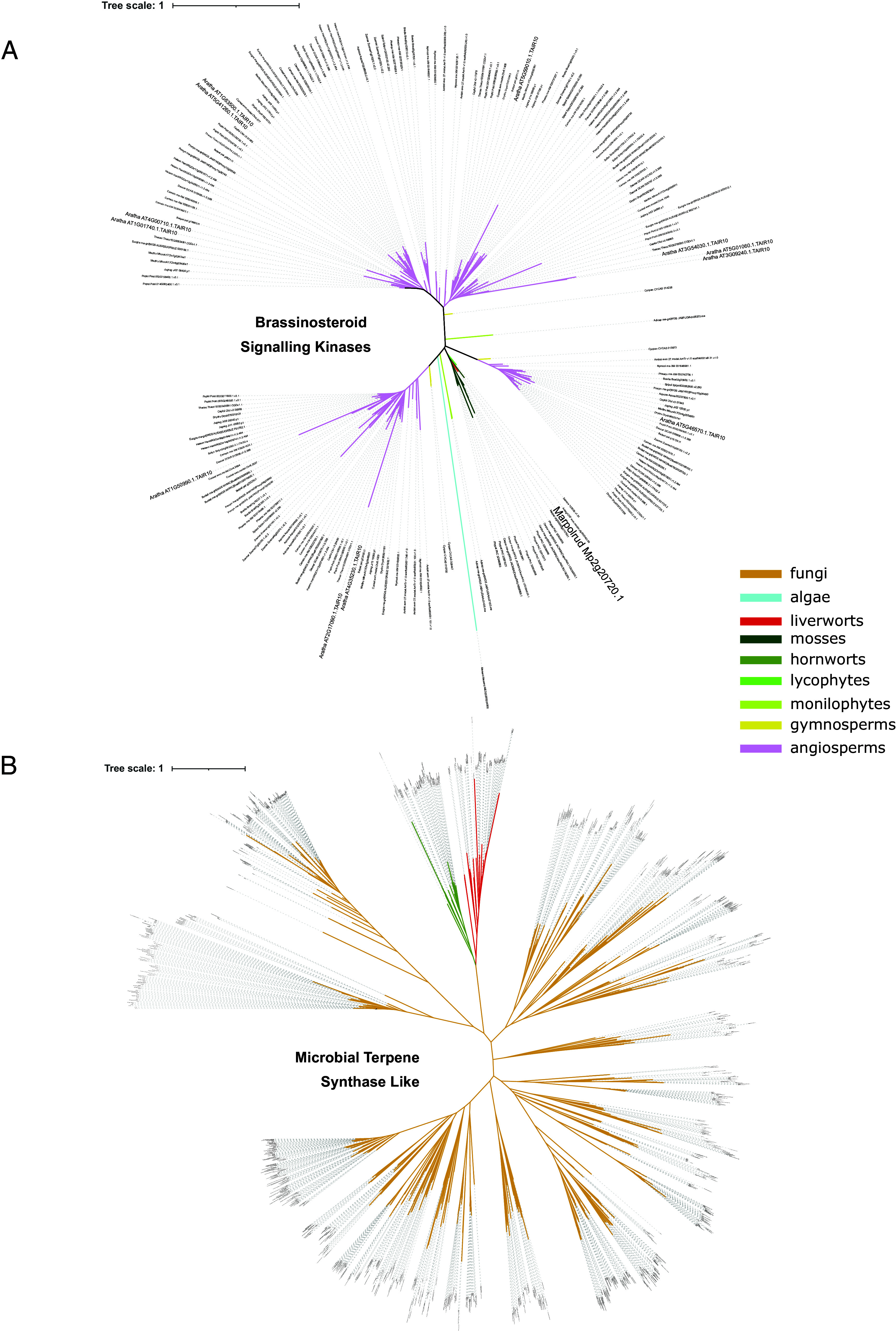
Phylogeny of the orthologs of the main GWAS candidate genes: the RLK Mp2g20720 and the five MTPSL genes *Mp6g04580, Mp6g04590, Mp6g04605, Mp6g04610*, and *Mp6g04630*. (*A*) Orthologs of the RLK GWAS candidate from chromosome 2 in Viridiplantae. This tree was computed with the substitution model Q.plant+R7 and has a log-likelihood of −59,286.8768. (*B*) Orthologs of the MTPSL GWAS candidates from chromosome 6 in Viridiplantae and fungi. This tree was computed with the substitution model JTT+F+R10 and has a log-likelihood of −271,237.3539.

In particular, the GWAS identified five genomic regions associated with symptom development (brown area), corresponding to 16 genes (Fig. 4*A*). The peaks were distributed across the autosomes. On chromosome 1, an association signal was found overlapping a methyltransferase and the functional regulatory region of a thioredoxin (Mp1g00650). Thioredoxins are involved in plant immunity through their reductase activity on cysteine residues that function as signaling switches, as well as their role in ROS detoxification (35). Interestingly, this thioredoxin is downregulated following infection in Tak-1 but not in CA. On chromosome 2, a strong association signal was detected near genes encoding a tRNA synthetase (Mp2g20730) and a receptor-like kinase (Mp2g20720). At this locus, four of the most susceptible accessions share an alternative allele, potentially coupled with a small deletion, represented by missing SNP data (Fig. 4*B*). This receptor-like kinase is the proto-ortholog of the 12 brassinosteroid signaling kinases from *A. thaliana* (Fig. 5*A*), suggesting a possible role in brassinosteroid-mediated immunity, a pathway conserved across land plants (36).

Another association signal was found on chromosome 5, surrounded by two genes. One encodes an Orotidine 5’ decarboxylase (Mp5g15200) that usually participates in the synthesis of pyrimidine nucleotides. The other gene encodes an atypical kinase ABC1K (Mp5g15210), from a clade only found in algae, bryophytes, and lycophytes, that has a common ancestor with the ABC1K12 mitochondrial family (37), whose role is not well defined. Interestingly, this gene is under selective sweep in Marchantia (top 14% of genes with low values of Fay and Wu’s *H*) (26).

Further associations were found on chromosome 6 where a cluster of five microbial terpene synthase genes (MTPSL, Mp6g04580, Mp6g04590, Mp6g04605, Mp6g04610, and Mp6g04630), is associated with variations in symptom severity. Some of the accessions with the largest area of browning in response to the pathogen display a common haplotype (bottom of the SNP matrix Fig. 4*C*), with missing data in the SNP calling indicative of a potential deletion of this region in these accessions. Although none of the MTPSL-encoding genes from this cluster are deregulated in response to *C. nymphaeae*, the fifth one was upregulated in response to *P. palmivora* (15). This cluster of terpene synthases, likely from the functional group III of monoterpene synthases (38), belongs to one of the two families of microbial terpene synthases present in Marchantia, which is thought to have originated from a horizontal gene transfer from fungi (39). Phylogenetic analyses conducted on a much larger dataset than used previously (39) further confirm this hypothesis and position the HGT between an ancestor of the extant Dikarya fungi and the most recent common ancestor of the bryophytes (Fig. 5*B*). One of the drivers of tandem gene duplications is transposon activity. To determine whether such a mechanism could have been involved in the evolution of the MTPSL cluster, we searched for signs of transposons in the surrounding genomic region. The same transposon residues were observed flanking each gene of the MTPSL cluster, suggesting that this family of 13 fungal terpene synthases was probably duplicated via transposon activity after its HGT from fungi (*SI Appendix*, Fig. S3). This mechanism could explain the substantial structural variation observed in this region of chromosome 6. To further test the importance of this locus for resistance, we attempted to delete the entire cluster. While chimeric individuals harboring the deletion were identified, gemmae derived from these thalli were all genotyped as wild-type, preventing stable line generation and functional analysis (*SI Appendix*, Fig. S4).

Another significant locus was found on chromosome 8, where an association region was flanked by an unannotated gene (Mp8g05260) and the gene MpLDPS2 (Mp8g05270). Mp*LDPS2* encodes a protein bearing a 1,8-cineole synthase domain, known to convert geranyl pyrophosphate, the precursor of monoterpenes, into 1,8-cineole (also known as eucalyptol). MpLDPS2 also contains a lipid droplet-associated protein (LDPS) domain involved in the formation of lipid droplets. These organelles not only store lipids but also contain enzymes that produce specialized metabolites, such as terpenes in bryophytes (40). Interestingly, the association peak on chromosome 8 is due to the balanced coexistence of two alleles, the minor allele being present in 34 accessions (i.e., 44% of all accessions studied), among which 16 display severe browning symptoms (Fig. 4*D*). The two candidate regions identified by the GWAS, MTPSLs and LDPS2, are both pointing to a potential role for terpenes in the immune defenses of Marchantia, consistent with their known antimicrobial and antifungal activities (41).

Together, these findings, identified through the first GWAS analyses in bryophytes, suggest a complex genetic basis for quantitative resistance to *C. nymphaeae* in *M. polymorpha*, involving receptor-Like kinases, ROS homeostasis, cell wall reinforcement, proteasome regulation, and secondary metabolism (notably phenylpropanoid and terpene pathways). These results highlight the likely importance of both core immune components and specialized metabolites in Marchantia defense.

## Discussion

Since the emergence of *M. polymorpha* as a suitable model for exploring evoMPMI (42) at the scale of land plants, most studies have focused on describing the conservation of immune mechanisms (for review refs. 3, 43, and 44). However, lineage-specific immune mechanisms remain underexplored. To address this gap, we investigated the naturally occurring pathosystem involving the filamentous hemibiotrophic pathogen *C. nymphaeae* and the genetic diversity within the *M. polymorpha* collection (26). Our transcriptomic analysis revealed significant overlap with the response of *M. polymorpha* to *P. palmivora* infection, with 80% of the DEGs shared between the two datasets (15). This finding suggests a core response to filamentous pathogens and supports the conservation of immune mechanisms between *M. polymorpha* and angiosperms, as previously suggested, highlighting a core set of genes involved in plant defense. This includes PR proteins, enzymes of the phenylpropanoid pathway, and transcription factors (WRKY, NAC, AP2/ERF).

Many of the gene families enriched among the upregulated genes such as LOX, NLR, and peroxidases, belong to the accessory genome of *M. polymorpha* (26). This reinforces the proposed role of the accessory genome in adaptation to environmental stresses. Our analyses also identified accession-specific genes upregulated in response to *C. nymphaeae*, but their precise role in defense against this pathogen requires further investigation.

Through GWAS, we identified terpene metabolism as a potential contributor to resistance to microbial infections in *M. polymorpha*. Although the underlying mechanisms remain to be validated, terpenes are well known for their role in biotic stress responses (45). Liverworts, including *M. polymorpha*, exhibit a remarkable diversity of terpenes, with approximately 1,600 identified compounds, including unique sesquiterpenes (45, 46). This diversity of terpenes is driven by terpene synthases (TPS) which produce a wide range of terpenes from common substrates (47). *M. polymorpha* harbors both typical plant TPS and microbial-like TPS, the latter sharing similarities with bacterial and fungal enzymes (39). The dual presence of TPS types is characteristic of bryophytes and other nonseed plants, while MTPSL are absent in seed plants (39). The *M. polymorpha* Tak-1 genome encodes 32 MTPSL genes out of a total of 39 terpene synthase genes, including both bacterial-like MTPSL and fungal-like MTPSL genes (16). In contrast, no MTPSL genes have been identified in the moss *P. patens* (48), and the genomes of the sequenced hornworts (*A. agrestis* and *A. punctatus*) contain only 6 and 7 MTPSL genes, respectively (49, 50). This variation may explain the observed differences in terpene diversity across bryophytes and other lineages, illustrating potential differences in their immune mechanisms.

In *M. polymorpha*, terpenes accumulate in oil bodies that are liverwort-specific structures where MTPSL enzymes localize (51, 52). Mass spectrometry analysis of oil body content revealed that sesquiterpenes and aromatic bisbibenzyls such as Marchantin A, accumulate in these organelles (53). This supports a functional link between oil bodies and the exceptional terpene diversity in liverworts. Consistent with this, species lacking oil bodies produce no sesquiterpenes and possess less complex biosynthetic pathways (39, 51). Although oil bodies are known to contribute to defense against herbivores (18, 54), the metabolites they store also exhibit antimicrobial and antifungal properties (45, 51). A potential role of oil body-stored terpenes in immunity, beyond defenses against herbivory, can therefore be hypothesized. Besides terpenes, the accumulation of the liverwort-specific anthocyanin Riccionidin A is associated with resistance against the oomycete *P. palmivora* (15).

MTPSL genes in *M. polymorpha* originated from horizontal gene transfer (HGT) events involving bacteria and fungi. A comprehensive analysis including bryophytes revealed two major HGT events involved in plant adaptation to terrestrial environments: one at the origin of streptophyte algae and another correlating with the plant terrestrialization. Interestingly, one-third of HGT-derived genes are associated with specialized metabolism (55), suggesting that MTPSL likely contributed to adaptation to terrestrial environments by enabling the synthesis of “specific” terpenes which may have been essential for defense.

Beyond MTPSL, our analysis identified enrichment of fungal fruit body lectin and Aerolysin/ETX pore-forming domains among the most significantly upregulated genes in the *M. polymorpha* CA accession. These genes were also acquired through HGT (26, 55) and may play a role in *M. polymorpha* immunity. For example, injection of fungal fruit body lectin from *M. polymorpha* significantly increased the mortality rate of the diamondback moth (55). A third example of an HGT-acquired gene with relevance in plant defense is the GH88 domain, enriched among upregulated genes in response to *C. nymphaeae*.

Altogether, these findings suggest that HGT has significantly contributed to the evolution of both core land plant and bryophyte-specific immunity landscapes, providing new metabolic and structural tools for environmental adaptation and defense.

To conclude, the integration of transcriptomic analysis and GWAS has identified both core and Marchantia-specific immune mechanisms, highlighting the likely pivotal roles of horizontal gene transfer and specialized metabolism in this process. Further functional investigations, such as allele swapping and targeted gene knockouts – unsuccessfully attempted here—are required to understand the precise functions of these genes and loci in *M. polymorpha* immunity.

## Online Methods

### Identification of *C. nymphaeae* Growth and Infection.

The *Colletotrichum spp.* strain used in this study was provided by J. Nelson (23). Six sequences, classically used to identify Colletotrichum species (27) (*SI Appendix*, Table S1), were amplified.

### Phenotyping of Various Accessions in Response to *C. nymphaeae*.

Accessions of *Marchantia polymorpha* were cultivated from gemmae under axenic conditions and grown on ½- strength Gamborg B5 medium (Sigma, G5396), pH 5.7, 1.4% Agar-Agar (Sigma, A7921) under a long-day photoperiod (16 h light at 22 °C/8 h dark at 20 °C), with 60-80 μE light intensity. Inoculation was performed by depositing a 10 µL droplet of a suspension of *C. nymphaeae* (10^4^ spores/mL), at the base of each thallus.

Images were analyzed using the software Image-Pro Plus© for the studied time points.

### Scanning Electron Microscopy (SEM).

Samples were fixed in a 0.05 M sodium cacodylate solution (pH 7.2) containing 2.5% glutaraldehyde, then dehydrated through a graded ethanol series. Images were acquired using a scanning electron microscope (Quanta 250 FEG, FEI) at 5 kV, with a spot size of 3 and a working distance of 10 mm.

### Bright-Field Microscopy.

For optical microscopy, 110-µm-thick sections of fresh *M. polymorpha* thalli were collected at various time points after inoculation with *C. nymphaeae*. The thallus sections, including rhizoids, were stained with WGA-FITC (50 µg/mL in PBS), washed three times with PBS, and mounted on glass slides in a drop of PBS. Observations were performed using bright-field microscopy or epifluorescence microscopy with an inverted microscope (Nikon Eclipse TI) equipped with a color CMOS camera DS-Ri2 and controlled by Nikon NIS software. A 10× (N.A 0.3) objective was used. Alternatively, samples were analyzed using a confocal microscope (LEICA SP8) at the FRAIB imaging platform.

### Confocal Microscopy.

Confocal images of rhizoids and *Colletotrichum* were acquired using a spectral confocal laser scanning system (SP8, Leica, Germany) equipped with an upright microscope (DMi8, Leica, Germany).

### RNA-Seq Experiment.

Three-week-old thalli from CA and Tak-1 were mock inoculated (water) or with a suspension of *C. nymphaeae*.

Total RNA was extracted from ~100 mg of ground thalli using the DirectZol kit (Ozyme, R2052) following the manufacturer’s instructions and sent for sequencing to Genewiz (Leipzig, Germany). Illumina libraries were prepared and sequenced on a NovaSeq platform using 150 bp paired-end reads, generating approximately 20 million reads per sample.

### Expression Analysis.

The raw reads were processed and mapped to their representative genome (Marchantia polymorpha Tak1 v6 and Marchantia polymorpha CA v1) with Nextflow v21.10.6 (56) and the nf-core/rnaseq r3.9 (57) pipeline, using the --skip_qc --aligner star_salmon, and --remove_ribo_rna options. Differentially expressed genes were identified using the edgeR package (58) in R v4.4.0, separately on each accession.

### IPR Term Enrichment.

Enrichment analyses were performed on the different categories of differentially expressed genes with the FUNC-E package v2.0.1 (59) with an enrichment cutoff of 0.01.

### Estimation of Accession Phenotypic Means.

A linear model was applied to the data for each phenotypic variable (except the thallus area preinoculation, which was used as a covariate) in order to estimate the accession means according to various confounding effects (effect of the experimental batch, the phytotron, and the area of the thallus preinoculation).

### Genome-Wide Association Study.

GWAS analyses were performed using the mixed linear model implemented in gemma software -v0.98.1- (60) on a dataset of 2,141,087 SNPs with minor allele frequencies of 0.05 and a maximum of 15 accessions with missing data per site, on a set of 77 phenotyped accessions from the subspecies *ruderalis*. To estimate the SNP effects and their significance, the model used a centered kinship matrix as a covariate with random effect, and a Wald test.

### Phylogenies of Candidate Genes.

Phylogeny for the MTPSL genes (Mp6g04580, Mp6g04590, Mp6g04605, Mp6g04610, and Mp6g04630) and for the RLK GWAS candidate from chromosome 2 (Mp2g20720) was determined by BLASTp+ v2.12.0 (61) (maximum of 2,000 target sequences and E-value of 10^−5^) against a database of Viridiplantae genomes (Dataset S10), a database with nonangiosperm transcriptomes from the 1KP initiative (62), a database with fungal genomes from MycoCosm [(63); last time consulted 02/2019], and the nonredundant (nr) database from the NCBI.

## Supplementary Material

Appendix 01 (PDF)

Dataset S01 (XLSX)

Dataset S02 (XLSX)

Dataset S03 (XLSX)

Dataset S04 (XLSX)

Dataset S05 (XLSX)

Dataset S06 (XLSX)

Dataset S07 (XLSX)

Dataset S08 (XLSX)

Dataset S09 (TXT)

Dataset S10 (XLSX)

## Data Availability

RNAseq data have been deposited in SRA (PRJNA1218021) (31).
