# Supplementary material for: Plant–fungi interactions in Marchantia polymorpha are associated with horizontal gene transfer and terpene metabolism
Source: Proc Natl Acad Sci U S A. 2026 Feb 4;123(6):e2532723123. doi: 10.1073/pnas.2532723123 (PMC12890914; doi:10.1073/pnas.2532723123)
Supplement: Supplementary file 1 — Appendix 01 (PDF) [file pnas.2532723123.sapp.pdf]

Supplemental information

Supplementary material and method

Identification of *C. nymphaeae* growth and infection

Six sequences including *Glyceraldehyde 3-phosphate dehydrogenase* (*GAPDH*), Histone H3 (*HIS3*); Chitin synthase (*CHS-1*), *Actin* (*ACT*), *b-tubulin-2* (*TUB2*), and the internal transcribed spacers (ITS1 and ITS2) were amplified. The amplified fragments were Sanger sequenced and BLAST searches conducted against the nr database. For each sequence, the first hit value corresponded to the *C. nymphaeae* species with percentages of identity comprised between 92.2 to 99.49 % and e-value ranging from $5e^{-113}$ to 0, confirming the species identification.

Phenotyping of various accessions in response to *C. nymphaeae*

For each accession, to ensure the formation of uniform thalli 36 similar sized gemma were propagated, and distributed across four square plates (12*12 cm). From the four plates, two pairs were formed with each pair placed in a different phytotron that maintained identical conditions. After three weeks of development, one plate from each pair was used for inoculation, while the other remained unmanipulated to prevent contamination. Following inoculation, the pairs of plates were placed back into their respective phytotrons. Symptoms were monitored daily through scans (EPSON GT20000). After each scan, the pairs were randomly rearranged within their phytotron. The *Marchantia polymorpha* collection was split into eight experimental batches during phenotyping, and two accessions were used as internal controls: CA and Tak-1, respectively resistant and susceptible to *C. nymphaeae*. Two weeks before inoculation, 200 µl of a 50% glycerol stock were spread on Mathur medium. Spores were collected by adding 5 mL of sterile water and scraping the mycelium.

A macro (Dataset S9) was used to automate the analysis and determine the thallus area. Symptomatic areas were manually adjusted by applying “Split Object” or “Draw/Merge Objects”.

Scanning Electron Microscopy (SEM)

Critical-point drying was performed using liquid CO_2. The samples were mounted on an observation plate with the rhizoids facing upwards and grounded using conductive silver paint. The samples were sputter-coated with platinum before imaging.

Bright field microscopy

Thalli were embedded in 5% low–melting-point agarose and sectioned using a vibratome (VT1000S; Leica, Rueil-Malmaison, France). The fluorescence of WGA-FITC was observed using a GFP SMO filter set (excitation: 472/30 nm; dichroic: 495 nm; emission: 520/35 nm),

while the autofluorescence of the cell walls was detected using a CY3 filter set (excitation: 543/22 nm; dichroic: 560 nm; emission: 590/40 nm). Alternatively, samples were analyzed using a confocal microscope (LEICA SP8) at the FRAIB imaging platform.

Confocal microscopy

Observations were performed with a 10× dry objective (HC PL FLUOTAR, N.A. 0.30). An Argon laser emitting at 488 nm was used to detect WGA-FITC fluorescence in the range of 500–540 nm. Additionally, a laser diode emitting at 405 nm was used to visualize rhizoid cell walls stained with calcofluor, with fluorescence collected in the range of 410–460 nm. Images were acquired in sequential mode to prevent inter-channel crosstalk. Fluorescence overlay images were generated by projecting 20–30 confocal planes acquired along the z-axis with a 4-µm increment between focal planes, creating a 3D projection.

RNA-Seq experiment

For each kinetic timepoint, the meristematic zones were harvested separately from the rest of the thallus, as these zones are symptom free and believed to exhibit a distinct response to infection. The samples were then frozen in liquid nitrogen, grounded and stored at -70°C until RNA extraction.

Annotation of the CA genome

The genome from CA 1was soft-masked using Earlgrey v4.3.0 (2) and the structural annotation was conducted using BRAKER v3.0.7 pipeline (3,4,5, 6,7, 8,9 ,10). BRAKER2 was run with –prot_seq –bam --gff3 and –busco_lineage options. In ETPmode the GeneMark-ETP pipeline generates hints based on the alignment of RNAseq reads and on the protein database supplied which enables AUGUSTUS training. The prediction of protein coding genes is a combination of AUGUSTUS and GeneMark-ETP predictions. The OrthoDB input proteins used by ProtHint is a combination of https://v100.orthodb.org/download/odb10_plants_fasta.tar.gz and proteins from seven species (Anthoceros agrestis cv. BONN, Anthoceros agrestis cv. OXF, Anthoceros punctatus (11), Ceratodon purpureus strain R40 (NCBI GCA_014871385.1), Marchantia paleacea (12), Marchantia polymorpha ssp. ruderalis TAK1 (https://marchantia.info/download/MpTak_v6.1/), Physcomitrium patens (13) and Sphagnum fallax (14). The completeness of the prediction was assessed with Compleasm (15) against the viridiplantae odb10 (n=425). The predictions were functionally annotated with interproscan-5.64-96.0 (16,17) with options –iprlookup and –goterms. The gene-to-gene correspondence between Tak-1's and CA's genome was determined combining the information from a collinearity comparison of the two genomes with MCscanX (18) and the mapping of Tak-1's annotation on CA's genome with Liftoff v1.6.3 (19).

Expression analysis

The nextflow workflow used bedtools v2.30.0 (8), gffread v0.12.1 (7), star v2.7.10a (20), picard v2.27.4, salmon v1.5.2 (21), SummarizedExperiment v1.20.0 (22), tximeta v1.8.0 (23samtools v1.15.1 (24), sortmerna v4.3.4 (), stringtie v2.2.1 (5), trimgalore v0.6.7 and usc v377. Low-expressed genes with less than ten reads across each class of samples were removed and gene counts were normalized by library size and using the trimmed mean of M-values normalization method (26). DEGs were estimated by pairwise comparisons between infected and mock inoculated samples at the same stage of infection. Differentially expressed genes (adjusted p-value ≤ 0.05 and absolute logFC [log2 fold change] ≥ 1) were used to perform hierarchical clustering of samples. Heatmaps for the DEGswere generated using R ComplexHeatmap package (27) using variance-stabilized counts median-centered by gene.

IPR term enrichment

Different categories of differentially expressed genes were considered for the enrichment analyses (in each accession, at each timepoint, up and down regulated genes), except from the ones that had a low number of differentially expressed gene (0 dpi, down regulated in CA).

Estimation of accessions phenotypic means

Phenotypic data on the thallus area, browning area of the thallus, and on the ratio between both, in inoculated plants at 0 and 6 dpi, were used to perform a genome wide association study. First, for each accession, outlier individuals (with their value being higher or lower than 1.5 times the interquartile range) for at least two phenotypic variables were discarded. Then, a linear model was applied on the data for each phenotypic variable (except the thallus area pre-inoculation, that was used as a covariable) in order to estimate the accessions means according to various confounding effects (effect of the experimental batch, of the phytotron and of the area of the thallus pre-inoculation). For the Tak-1 (susceptible) and CA (resistant) accessions that were present in each experimental batch, the linear model is as follows: $phenotype_{ijkl} = \mu + accession_i + phytotron_j * batch_k + preinoc\_thallus\_area_l + \varepsilon_{ijkl}$, where μ is the overall mean, “accession” corresponds to the difference between Tak-1 and CA accessions, “phytotron” and “batch” account for the effect of the two phytotrons used and the experimental batch in which each plant was grown, “preinoc_thallus_area” is a quantitative covariate accounting for the initial thallus size of each plant, and ε is the residual term. This allows to get adjusted means for CA and Tak-1, that will be used as their phenotypic values in the GWAS. In order to use Tak-1 as an internal control of the experimental batches involving all other *M. polymorpha* accessions, another linear model is implemented that only considers the experimental batch and the initial thallus size of each Tak-1 plant before inoculation:

$phenotype_{ij} = \mu + batch_i + preinoc\_thallus\_area_j + \varepsilon_{ij}$. The estimated "batch" effects are then used as a covariable ($TAK1\_ctrl$) for the other accessions in the same experimental batch, in the following linear model: $phenotype_{ijkl} = \mu + accession_i * phytotron_j + TAK1\_ctrl_k + preinoc\_thallus\_area_l + \varepsilon_{ijkl}$. For each phenotype analysed, adjusted means of each *M. polymorpha* accession is estimated, and then serve as inputs in the GWAS.

Genome wide association study

SNP *P*-values from GEMMA were processed using a local score approach (28,29) to help detect robust loci across analyses. The local score is a cumulative score that takes advantage of local linkage disequilibrium among SNPs. This score, defined as the maximum of the Lindley process over a SNP sequence (i.e., a chromosome), was calculated using a tuning parameter value of ξ=2, as suggested by simulation results (28). Chromosome-specific significance thresholds (α =5%) were estimated using a resampling approach. The R scripts used to compute the local score and significance thresholds are available at https://forge-dga.jouy.inra.fr/projects/local-score/documents. All the significant local score peaks coordinates were extracted, and they were annotated with their overlapping (when existing), downstream and upstream genes.

Phylogenies of candidate genes

The resulting proteins were aligned with muscle5 v5.1 (30) and trimmed with trimAl v1.4 (31) to discard positions with more than 60% of gaps. The phylogenetic tree was computed with IQ-TREE v2.1.2 and the best-fitting evolutionary model selected using modelFinder according to the Bayesian Information Criteria. Branch support was estimated with 10 000 replicates of both SH-like approximate likelihood ratio and ultrafast bootstrap. For the receptor-like kinase (Mp2g20720), the same tools were used but the sequence research was only performed against the custom database of plant genomes. For the GH88 domain, sequence research and alignment were performed the same way, but the phylogeny was performed with FastTree v2.1.11 (32) with the default options.

Supplementary file legends

Dataset S1: Detailed table of the Hierarchical clustering of Tak-1's significantly differentially expressed genes during *C. nymphaeae* infection (adjusted p ≤ 0.05; log2 fold change [logFC] ≥ 1) at 0 and 3 days post inoculation (dpi) (Figure 3.a). Variance stabilized row-centered counts are shown. Additional information on these genes is specified: gene symbol, functional annotation, pangenomic compartment of the genes, differential expression during the infection with *P. palmivora* (adjusted p-value ≤ 0.05; absolute log2 fold change [logFC] ≥ 1), and

differential expression under different single or cumulated abiotics stresses (N=nitrogen deficiency, S=salt, L=light, M=mannitol, C=cold, D=dark, H=heat).

Dataset S2: Full table of the functional enrichments (GO and IPR terms) on Tak-1 up and down regulated genes in response to *C. nymphaeae* at 3 dpi (enrichment cutoff of 0.01). The IPR enrichment were then curated to remove the redundant terms in order to produce the Figure 3.b.

Dataset S3: Table of the differential expression of all *M. polymorpha* genes in Tak-1 and CA at 3 and 6 dpi with *C. nymphaeae*. Information about the GWAS candidate genes, the genome environment association candidate genes and pangenomic compartment (Beaulieu et al. 2025), the cell cluster assignation by single cell RNAseq (Wang et al. 2023), the differential expression under *P. palmivora* infection (Carella et al. 2019), under *P. syringae* infection (Grenz et al. 2024) and under various abiotic stresses (Wen Tan et al. 2023), as well as the functional annotations are specified. Correspondence between CA and Tak-1 genes can lead to multiple lines for a single gene in one accession (but multiple corresponding genes in the other accession).

Dataset S4: Cross referencing of the single copy orthogroups differentially expressed in both *M. polymorpha* and *N. benthamiana* in response to *P. palmivora* (Carella et al. 2019) with *M. polymorpha* differentially expressed genes in response to *C. nymphaeae*, and functional enrichment on the genes commonly up regulated in *M. polymorpha* in response to both pathogens and in *N. benthamiana* in response to *P. palmivora*.

Dataset S5: Detailed table of the Hierarchical clustering of CA's significantly differentially expressed genes during *C. nymphaeae* infection (adjusted $p \leq 0.05$; log2 fold change [logFC] ≥ 1) at 0, 3 and 6 days post inoculation (dpi) (Figure 3.c). Variance stabilized row-centered counts are shown. Additional information on these genes is specified: gene symbol, functional annotation, pangenomic compartment of the genes, differential expression during the infection with *P. palmivora* (adjusted p-value ≤ 0.05; absolute log2 fold change [logFC] ≥ 1), and differential expression under different single or cumulated abiotic stresses (N=nitrogen deficiency, S=salt, L=light, M=mannitol, C=cold, D=dark, H=heat).

Dataset S6: Full table of the functional enrichments (GO and IPR terms) on CA up regulated genes at 3 and 6 dpi in response to *C. nymphaeae* (enrichment cutoff of 0.01). Not enough genes were down regulated to perform an enrichment on them. The IPR enrichment were then curated to remove the redundant terms in order to produce the Figure 3.d.

Dataset S7: List of genomic regions and genes associated with the four traits considered at 6dpi: thallus area of *C. nymphaeae* inoculated plants, thallus area of mock inoculated plants, symptoms area (brown) and ratio of symptoms area on thallus area (brown percentage). The genome wide association study was performed on 77 *M. polymorpha* ssp. *ruderalis* accessions.

Dataset S8: Macros used for *M. polymorpha* phenotyping.

Dataset S9: Species contained in a database of 261 genomes from all the main clades of land plants, and used for phylogenetic analyses.

<u>Supplementary Figures and Table</u>

Figure S1: Phenotypic variability in response to *C. nymphaeae* infection, within the *M. polymorpha* collection. *C. nymphaeae* infection causes maceration of the thallus resulting in browning. In the most susceptible accessions, white hyphae are visible on the surface of the thallus. Nor-E : Norwich-E, Tou-C : Toulon-C, Gil-1: Gilesgate-1, Voe-B : Voewood-B, Cam-2 : Cambridge-2, Tak-1: Takaragaike-1, Bul-B: Bulan-B.

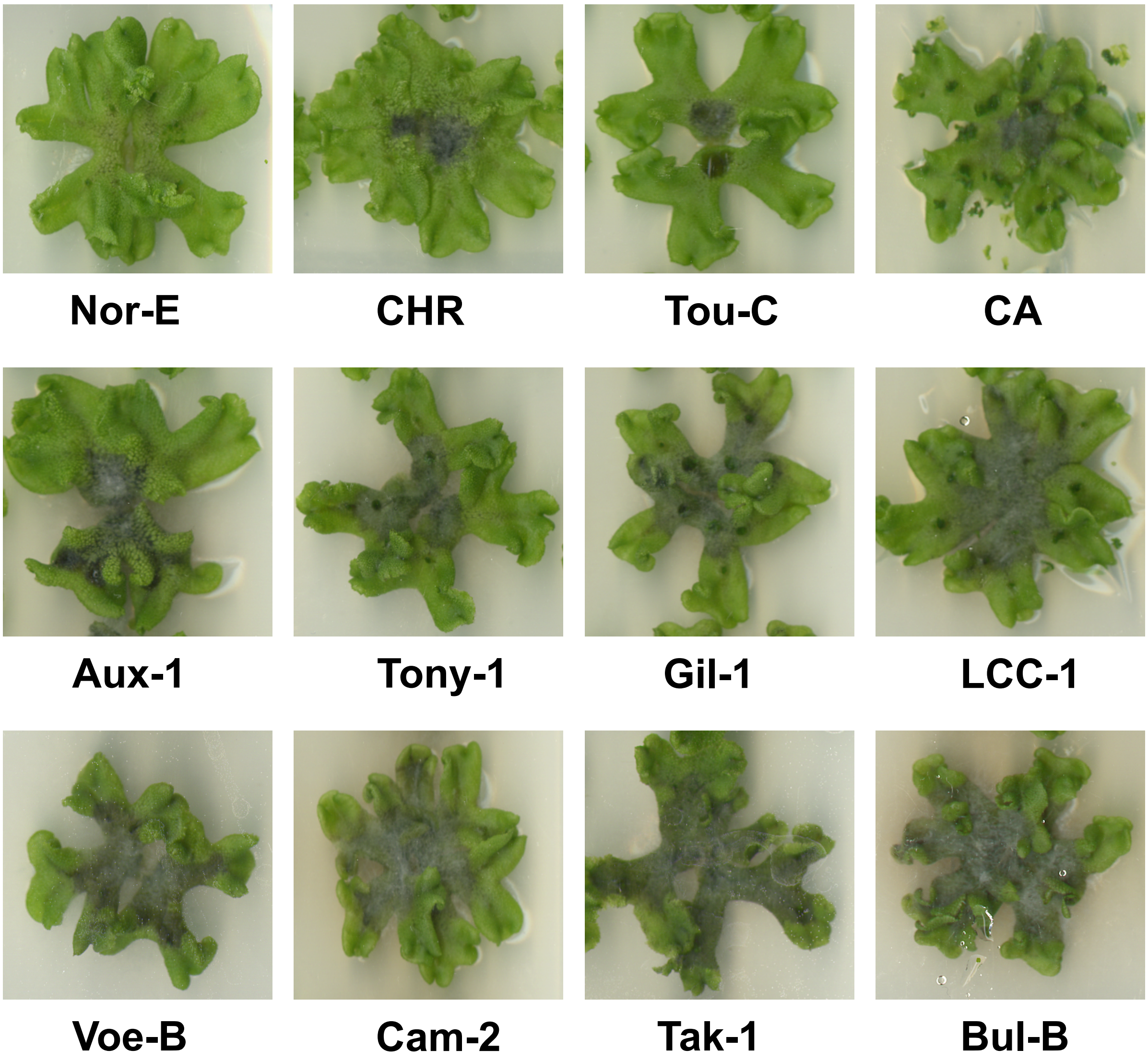

Figure S2: Phylogeny of *M. polymorpha'*s GH88 gene (Mp3g19320). This tree was computed with FastTree.

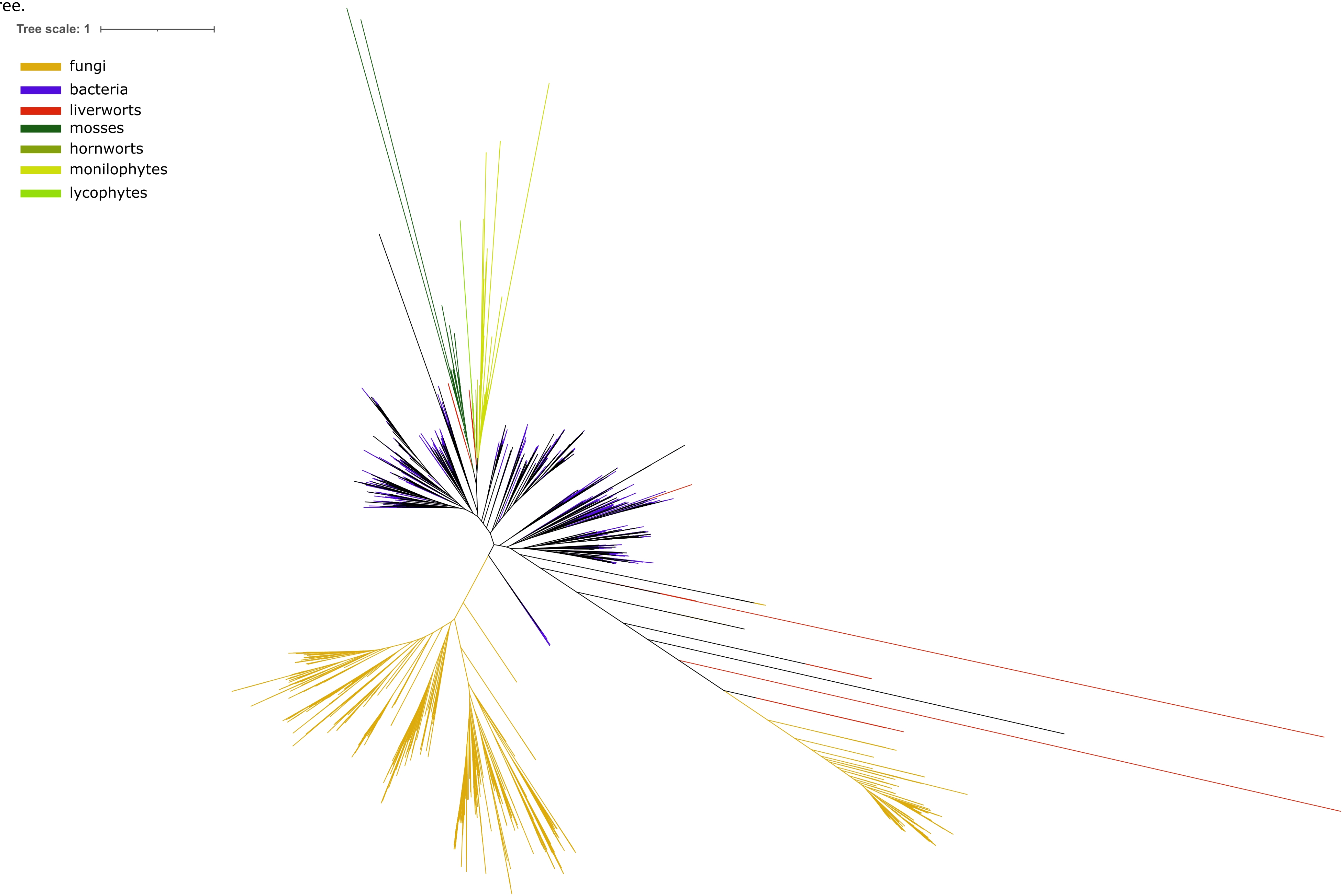

Figure S3: Extract of *M. polymorpha*'s genome browser showing the transposon signature flanking all genes in the MTPSL cluster below the GWAS peak on chromosome 6.

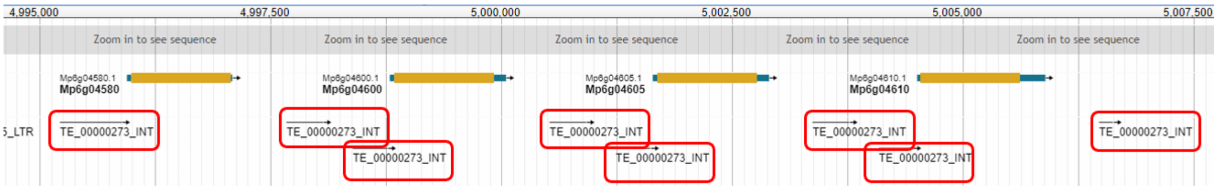

Figure S4. Tentative of CRISPR mutagenesis on *Mp2g20720* and MTLS cluster in CA. CA plants were transformed with a T-DNA containing Cas9 under the control of et MpEF1a promoter and four sgRNAs targeting *Mp2g20720* (1: TTGGCGGGCTGGCCCTGTAG-NGG, 2: CTGCTTACAGTCGAAGCCCA-NGG, 3: CTTCTCCAATTGCAAACAGA-NGG, 4: TGCCACAACATATAATGTAA-NGG) or the MTPSL cluster locus (1: GTTAACTAGCAACTAATTGG-NGG, 2: TCATCTTGTGATACAAATGA-NGG, 3: AGTATTGGTAGGACACAGAG-NGG, 4: GCAAGACGACACGCCTGAGG-NGG). CRISPR-induced full gene deletions were screened by PCR for *Mp2g20920* (Fw: GTGCTCTGTGTTGCGAGAAA, Rv: ACAGGATCCAACCCACCAAT, Ta = 60 °C) or MTPSL (Fw: GCAAAGGCGATATGTCTCTGG, Rv: TGCGATCAAGGTACCGGAAT, Ta = 58 °C). Some mutants bands were identified and confirmed by capillary sequencing. Two progenies of each mutant were tested by PCR in same conditions as for T1 (apart from an extension time of 1 minute 30 secondes for *Mp2g20920* in T2, instead of 30 secondes used in T1 genotyping, amplifying the WT sequence), but the mutant band could not be seen. We concluded that the mutations observed in T1 derive from some somatic cells, and were not inherited stably in T2.

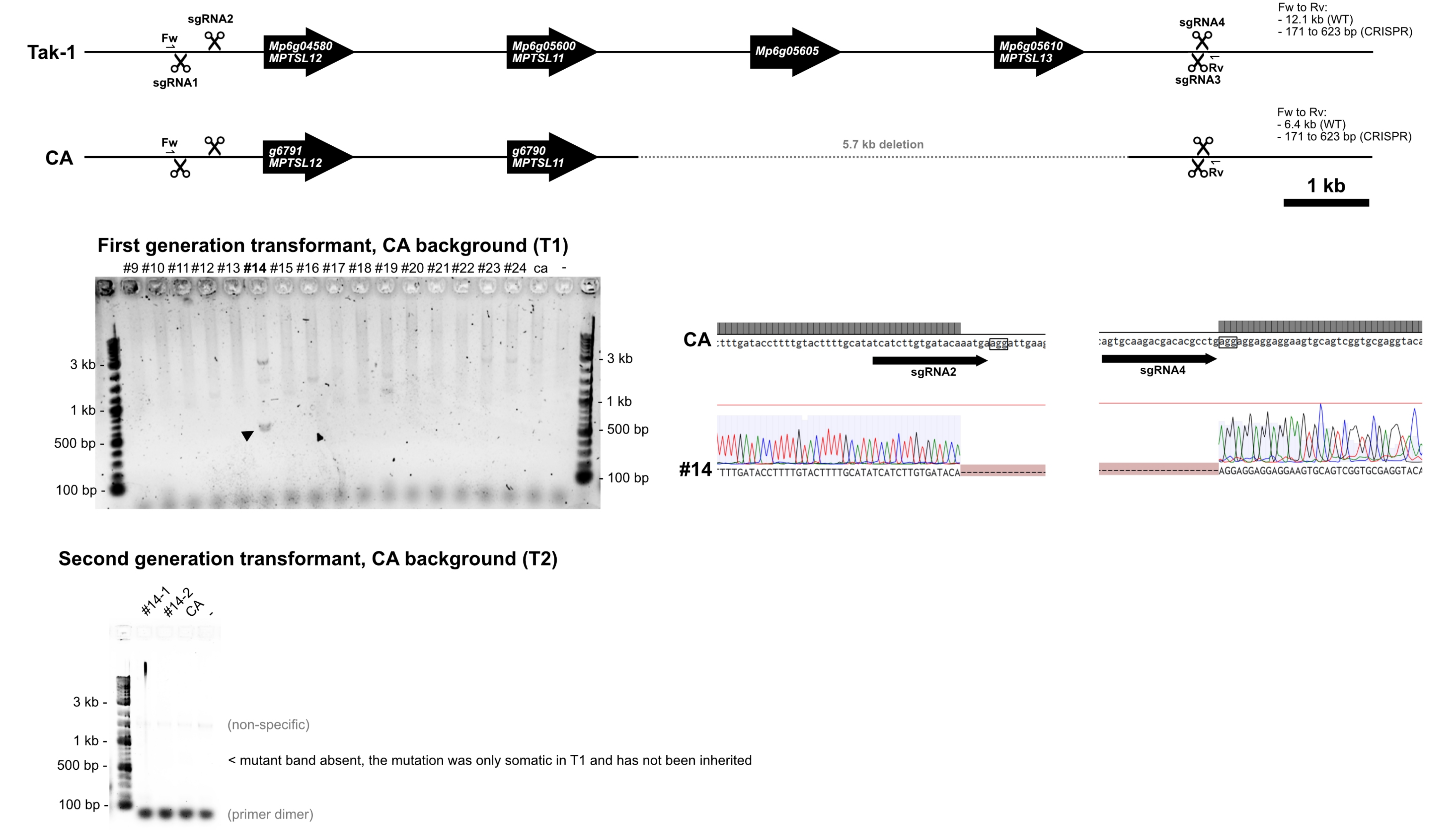

Supplementary Table 1 : Primers used to identify *Colletrotrichum spp*. (based on Damm *et al*., 2014)

Name of targeted sequence	Forward (F) /Reverse (R)	Primer sequence
**ITS-1**	F	CTTGGTCATTTAGAGGAAGTAA
	R	TCCTCCGCTTATTGATATGC
**GAPDH**	F	GCCGTCAACGACCCCTTCATTGA
	R	GGGTGGAGTCGTACTTGAGCATGT
**CHS1 (partial)**	F	TGGGGCAAGGATGCTTGGAAGAAG
	R	TGGAAGAACCATCTGTGAGAGTTG
**HIS3**	F	AGGTCCACTGGTGGCAAG
	R	AGCTGGATGTCCTTGGACTG
**ACT**	F	ATGTGCAAGGCCGGTTTCGC
	R	TACGAGTCCTTCTGGCCCAT
**TUB**	F	ACCCTCAGTGTAGTGACCCTTGGC
	R	ACCCTCAGTGTAGTGACCCTTGGC

References

1. Beaulieu, C., Libourel, C., Zamar, D. L. M., Mahboubi, K. E., Hoey, D. J., Greiff, G. R. L., Keller, J., Girou, C., Clemente, H. S., Diop, I., Amblard, E., Castel, B., Théron, A., Cauet, S., Rodde, N., Zachgo, S., Halpape, W., Meierhenrich, A., Laker, B.,. . . Delaux, P. (2025). The Marchantia polymorpha pangenome reveals ancient mechanisms of plant adaptation to the environment. *Nature Genetics*, *57*(3), 729-740. https://doi.org/10.1038/s41588-024-02071-4

2. Baril, T., Galbraith, J., & Hayward, A. (2024). Earl Grey : A Fully Automated User-Friendly Transposable Element Annotation and Analysis Pipeline. *Molecular Biology and Evolution*, *41*(4), msae068. https://doi.org/10.1093/molbev/msae068
3. Brůna, T., Lomsadze, A., & Borodovsky, M. (2020). GeneMark-EP+ : Eukaryotic gene prediction with self-training in the space of genes and proteins. *NAR Genomics and Bioinformatics*, *2*(2), lqaa026. https://doi.org/10.1093/nargab/lqaa026
4. Gabriel, L., Hoff, K. J., Brůna, T., Borodovsky, M., & Stanke, M. (2021). TSEBRA : Transcript selector for BRAKER. *BMC Bioinformatics*, *22*(1), 566. https://doi.org/10.1186/s12859-021-04482-0
5. Gabriel, L., Brůna, T., Hoff, K. J., Ebel, M., Lomsadze, A., Borodovsky, M., & Stanke, M. (2023). *BRAKER3 : Fully automated genome annotation using RNA-seq and protein evidence with GeneMark-ETP, AUGUSTUS and TSEBRA*. https://doi.org/10.1101/2023.06.10.544449
6. Kovaka, S., Zimin, A. V., Pertea, G. M., Razaghi, R., Salzberg, S. L., & Pertea, M. (2019). Transcriptome assembly from long-read RNA-seq alignments with StringTie2. *Genome Biology*, *20*(1), 278. https://doi.org/10.1186/s13059-019-1910-1
7. Pertea, G., & Pertea, M. (2020). GFF Utilities : GffRead and GffCompare. *F1000Research*, *9*, 304. https://doi.org/10.12688/f1000research.23297.2
8. Quinlan, A. R. (2014). BEDTools : The Swiss-Army Tool for Genome Feature Analysis. *Current Protocols in Bioinformatics*, *47*(1). https://doi.org/10.1002/0471250953.bi1112s47
9. Stanke, M., Schöffmann, O., Morgenstern, B., & Waack, S. (2006). Gene prediction in eukaryotes with a generalized hidden Markov model that uses hints from external sources. *BMC Bioinformatics*, *7*(1), 62. https://doi.org/10.1186/1471-2105-7-62
10. Stanke, M., Diekhans, M., Baertsch, R., & Haussler, D. (2008). Using native and syntenically mapped cDNA alignments to improve *de novo* gene finding. *Bioinformatics*, *24*(5), 637-644. https://doi.org/10.1093/bioinformatics/btn013
11. Li, F.-W., Nishiyama, T., Waller, M., Frangedakis, E., Keller, J., Li, Z., Fernandez-Pozo, N., Barker, M. S., Bennett, T., Blázquez, M. A., Cheng, S., Cuming, A. C., de Vries, J., de Vries, S., Delaux, P.-M., Diop, I. S., Harrison, C. J., Hauser, D., Hernández-García,

J., … Szövényi, P. (2020). Anthoceros genomes illuminate the origin of land plants and the unique biology of hornworts. *Nature Plants*, *6*(3), 259-272. https://doi.org/10.1038/s41477-020-0618-2
12. Radhakrishnan, G. V., Keller, J., Rich, M. K., Vernié, T., Mbadinga Mbadinga, D. L., Vigneron, N., Cottret, L., Clemente, H. S., Libourel, C., Cheema, J., Linde, A.-M., Eklund, D. M., Cheng, S., Wong, G. K. S., Lagercrantz, U., Li, F.-W., Oldroyd, G. E. D., & Delaux, P.-M. (2020). An ancestral signalling pathway is conserved in intracellular symbioses-forming plant lineages. *Nature Plants*, *6*(3), 280-289. https://doi.org/10.1038/s41477-020-0613-7
13. Lang, D., Ullrich, K. K., Murat, F., Fuchs, J., Jenkins, J., Haas, F. B., Piednoel, M., Gundlach, H., Van Bel, M., Meyberg, R., Vives, C., Morata, J., Symeonidi, A., Hiss, M., Muchero, W., Kamisugi, Y., Saleh, O., Blanc, G., Decker, E. L., … Rensing, S. A. (2018). The *Physcomitrella patens* chromosome-scale assembly reveals moss genome structure and evolution. *The Plant Journal*, *93*(3), 515-533. https://doi.org/10.1111/tpj.13801
14. Healey, A. L., Piatkowski, B., Lovell, J. T., Sreedasyam, A., Carey, S. B., Mamidi, S., Shu, S., Plott, C., Jenkins, J., Lawrence, T., Aguero, B., Carrell, A. A., Nieto-Lugilde, M., Talag, J., Duffy, A., Jawdy, S., Carter, K. R., Boston, L.-B., Jones, T., … Shaw, A. J. (2023). Newly identified sex chromosomes in the Sphagnum (peat moss) genome alter carbon sequestration and ecosystem dynamics. *Nature Plants*, *9*(2), 238-254. https://doi.org/10.1038/s41477-022-01333-5
15. Huang, N., & Li, H. (2023). compleasm : A faster and more accurate reimplementation of BUSCO. *Bioinformatics*, *39*(10), btad595. https://doi.org/10.1093/bioinformatics/btad595
16. Blum, M., Chang, H.-Y., Chuguransky, S., Grego, T., Kandasaamy, S., Mitchell, A., Nuka, G., Paysan-Lafosse, T., Qureshi, M., Raj, S., Richardson, L., Salazar, G. A., Williams, L., Bork, P., Bridge, A., Gough, J., Haft, D. H., Letunic, I., Marchler-Bauer, A., … Finn, R. D. (2021). The InterPro protein families and domains database : 20 years on. *Nucleic Acids Research*, *49*(D1), D344-D354. https://doi.org/10.1093/nar/gkaa977
17. Jones, P., Binns, D., Chang, H.-Y., Fraser, M., Li, W., McAnulla, C., McWilliam, H., Maslen, J., Mitchell, A., Nuka, G., Pesseat, S., Quinn, A. F., Sangrador-Vegas, A., Scheremetjew, M., Yong, S.-Y., Lopez, R., & Hunter, S. (2014). InterProScan 5 : Genome-scale protein function classification. *Bioinformatics*, *30*(9), 1236-1240. https://doi.org/10.1093/bioinformatics/btu031
18. Wang, Y., Tang, H., DeBarry, J. D., Tan, X., Li, J., Wang, X., Lee, T. -h., Jin, H., Marler, B., Guo, H., Kissinger, J. C., & Paterson, A. H. (2012). MCScanX : A toolkit for detection and evolutionary analysis of gene synteny and collinearity. *Nucleic Acids Research*, *40*(7), e49-e49. https://doi.org/10.1093/nar/gkr1293
19. Shumate, A., & Salzberg, S. L. (2021). Liftoff : Accurate mapping of gene annotations. *Bioinformatics*, *37*(12), 1639-1643. https://doi.org/10.1093/bioinformatics/btaa1016

20. Dobin, A., Davis, C. A., Schlesinger, F., Drenkow, J., Zaleski, C., Jha, S., Batut, P., Chaisson, M., & Gingeras, T. R. (2013). STAR : Ultrafast universal RNA-seq aligner. *Bioinformatics*, *29*(1), 15-21. https://doi.org/10.1093/bioinformatics/bts635
21. Patro, R., Duggal, G., Love, M. I., Irizarry, R. A., & Kingsford, C. (2017). Salmon provides fast and bias-aware quantification of transcript expression. *Nature Methods*, *14*(4), 417-419. https://doi.org/10.1038/nmeth.4197
22. Morgan M, Obenchain V, Hester J, Pagès H (2024). *SummarizedExperiment: A container (S4 class) for matrix-like assays*. doi:10.18129/B9.bioc.SummarizedExperiment, R package version 1.37.0, https://bioconductor.org/packages/SummarizedExperiment.
23. Love, M. I., Soneson, C., Hickey, P. F., Johnson, L. K., Pierce, N. T., Shepherd, L., Morgan, M., & Patro, R. (2020). Tximeta : Reference sequence checksums for provenance identification in RNA-seq. *PLoS Computational Biology*, *16*(2), e1007664. https://doi.org/10.1371/journal.pcbi.1007664
24. Li, H., Handsaker, B., Wysoker, A., Fennell, T., Ruan, J., Homer, N., Marth, G., Abecasis, G., Durbin, R., & 1000 Genome Project Data Processing Subgroup. (2009). The Sequence Alignment/Map format and SAMtools. *Bioinformatics*, *25*(16), 2078-2079. https://doi.org/10.1093/bioinformatics/btp352
25. Kopylova, E., Noé, L., & Touzet, H. (2012). SortMeRNA : Fast and accurate filtering of ribosomal RNAs in metatranscriptomic data. *Bioinformatics*, *28*(24), 3211-3217. https://doi.org/10.1093/bioinformatics/bts611
26. Robinson, M. D., & Oshlack, A. (2010). A scaling normalization method for differential expression analysis of RNA-seq data. *Genome Biology*, *11*(3), R25. https://doi.org/10.1186/gb-2010-11-3-r25
27. Gu, Z., Eils, R., & Schlesner, M. (2016). Complex heatmaps reveal patterns and correlations in multidimensional genomic data. *Bioinformatics*, *32*(18), 2847-2849. https://doi.org/10.1093/bioinformatics/btw313
28. Bonhomme, M., Fariello, M. I., Navier, H., Hajri, A., Badis, Y., Miteul, H., Samac, D. A., Dumas, B., Baranger, A., Jacquet, C., & Pilet-Nayel, M.-L. (2019). A local score approach improves GWAS resolution and detects minor QTL : Application to Medicago truncatula quantitative disease resistance to multiple Aphanomyces euteiches isolates. *Heredity*, *123*(4), 517-531. https://doi.org/10.1038/s41437-019-0235-x
29. Fariello, M. I., Boitard, S., Mercier, S., Robelin, D., Faraut, T., Arnould, C., Recoquillay, J., Bouchez, O., Salin, G., Dehais, P., Gourichon, D., Leroux, S., Pitel, F., Leterrier, C., & SanCristobal, M. (2017). Accounting for linkage disequilibrium in genome scans for selection without individual genotypes : The local score approach. *Molecular Ecology*, *26*(14), 3700-3714. https://doi.org/10.1111/mec.14141

30. Edgar, R. C. (2022). Muscle5 : High-accuracy alignment ensembles enable unbiased assessments of sequence homology and phylogeny. *Nature Communications*, *13*(1), 6968. https://doi.org/10.1038/s41467-022-34630-w
31. Capella-Gutierrez, S., Silla-Martinez, J. M., & Gabaldon, T. (2009). trimAl : A tool for automated alignment trimming in large-scale phylogenetic analyses. *Bioinformatics*, *25*(15), 1972-1973. https://doi.org/10.1093/bioinformatics/btp348
32. Price, M. N., Dehal, P. S., & Arkin, A. P. (2010). FastTree 2 – Approximately Maximum-Likelihood Trees for Large Alignments. *PLoS ONE*, *5*(3), e9490. https://doi.org/10.1371/journal.pone.0009490
